# Examining the stability and change in age-crime relation in South Korea, 1980–2019: An age-period-cohort analysis

**DOI:** 10.1371/journal.pone.0299852

**Published:** 2024-03-29

**Authors:** Yunmei Lu

**Affiliations:** Department of Sociology, University at Buffalo, SUNY, Buffalo, NY, United States of America; University of Alabama in Huntsville, UNITED STATES

## Abstract

The aggregate-level age-crime distributions in Western countries are predominantly right-skewed and adolescent-spiked. Based on Western data, Hirschi and Gottfredson (1983) asserted that this age-crime pattern is universally invariant across time and places. This study’s overall goal is to rigorously examine Hirschi and Gottfredson’s invariant premise within a non-Western country, focusing on the stability and change in the age-crime patterns of South Korea from 1980 to 2019. Specifically, two research questions are addressed: (1) whether the average age-arrest curves in South Korea diverge from the invariant premise after adjusting for period and cohort effects; (2) how period and cohort effects modify the age-arrest curves. To examine these questions, I applied the age-period-cohort-interaction model (APC-I) to analyze the official age-specific arrest statistics for various offense types from 1980 to 2019 in South Korea. Findings suggested that the age-crime patterns of homicide, assault, and fraud are characterized by spread-out distributions and advanced peak ages. After adjusting for period and cohort effects, most of the age-crime curves are still robustly divergent from the age-crime distributions found in Western countries. Cohort and period effects have modified the age-crime patterns, but arrests in South Korea are largely concentrated among midlife age groups older than 30. These results provide additional compelling evidence contesting Hirschi and Gottfredson’s invariance thesis, underscoring the substantial impact of country-specific processes, historical context, and cultural factors on the age-crime relationship.

## Introduction

The relationship between age and crime has been widely discussed in Western criminological literature for decades. Based on data in Western countries, Hirschi and Gottfredson [1] (HG hereafter) argued that the age-crime distribution is always right-skewed and adolescent-spiked across places and time. They further maintained that the ubiquity of this inverted J-shaped distribution is a “brute fact of criminology” and cannot be explained by extant sociological variables [1]. Instead, they asserted that age has a direct effect on crime, presumably through biological maturation. Many scholars today agree with the age-crime invariance thesis and consider it undisputed [2]. With a focus on establishing universal patterns driven by internal states, HG’s invariance thesis has been central in extensive research on adolescent risky behaviors across multiple areas in social and behavioral sciences, including evolutionary and developmental psychology, and some areas of neurobiology [3]. Specifically, this thesis has gained traction among scholars favoring evolutionary or biological interpretations of human development [3]. By contending that high levels of adolescent crime are a natural part of human development, the invariant adolescent-driven age-crime schedule questions the adequacy of numerous social theories that emphasize the importance of socio-cultural context in shaping human behaviors.

Other scholars, however, consider the matter of an invariant age-crime curve unsettled. Some scholars have presented variations in the parameters and shape of age-crime patterns for different offenses across historical periods in the U.S. [4–6]. The most prominent challenges emerged from Steffensmeier and colleagues’[3, 7, 8] recent studies on age-crime relationships in non-Western societies, including Taiwan, India, and South Korea (SK). In each of these countries, the age-crime patterns are more spread out with older peaks compared to those observed in U.S. For instance, they found most of the age-crime curves in SK from 2001 to 2010 are either left-skewed or nearly symmetrical with a peak between the 30s and 40s [8]. A cross-cultural comparative perspective is particularly valuable for validating the age-crime invariance thesis, as there is no justification to assume that innate biological factors involved in determining age effects would vary across countries. That is, there should be no international variation in the age-crime distribution if the age effects stem purely from biological or predetermined developmental factors. Therefore, utilizing international data and cross-cultural studies offers an intuitive and straightforward way to illustrate the significance of social context.

While providing important evidence to challenge the HG age-crime invariance thesis, Steffensmeier and colleagues’ studies are not without limitations. A major shortcoming of these studies is that they relied on *cross-sectional* data representing a specific period and therefore cannot provide definitive evidence of varying age-crime patterns. The age distribution of crime in a specific period is determined simultaneously by age as well as period and cohort effects [9, 10]. Period effects refer to social or historical changes that affect all age and cohort groups universally, such as a war or a natural disaster. If a period effect is present, it would increase or decrease the levels of crime rates for all age groups. Cohort effects refer to the unique experience shared by individuals born around the same time and encountering the same changes at a specific age, such as the baby boomers or the cohort growing up during the great depression who may face unique challenges in their life course [11, 12]. Different from period effects that are universal in all age groups, cohort effects may alter the shape of an age-crime pattern by changing the crime rate of a specific age (cohort) group in a specific period.

Very few prior studies have explicitly tested period and cohort effects on the age-crime relationship, but sociological literature has indicated that social or demographic changes may have differential impacts on various age groups thus altering the age-crime patterns [13, 14]. According to modernization theory, industrialization may lead to weakened social control and greater adult-adolescent generational separation, which contribute to higher levels of youth crime and a more right-skewed distribution [15]. In addition, demographic research has highlighted the importance of cohort replacement in shaping age-specific behaviors across time [16]. For instance, Easterlin [11] projected that a large cohort size might foster higher crime rates among adolescents because of heightened competition for limited economic resources and weakened societal control during adolescents’ formative years. In contrast, we would expect lower youth crime rates for smaller-sized cohorts. Studies in the U.S. have shown that period and cohort effects contribute to changes in age-crime patterns of some offense types [10, 17], but the effects have been relatively small.

In the past four decades, South Korea has undergone important societal transformations, including rapid economic growth and dramatic changes in the population’s age structure. As a part of the "East Asian Miracle", South Korea experienced swift economic development in the 1980s and 1990s, boasting an average annual GDP growth rate of 7% [18]. Despite facing challenges from regional and global economic recessions (e.g. Asian financial crisis in 1997 and the global economic recession in 2008), the country’s economy remained resilient and relatively strong throughout this period, largely due to its competitive industrial and services sectors [19, 20]. The demographic transformation in SK has been more dramatic than its economic development. Life expectancy at birth has increased from 66 in 1980 to 84 in 2020 because of a significant decline in mortality rates and advancements in medical science [20]. Meanwhile, the fertility rate has experienced a striking decline, plummeting from 2.8 births per woman in 1980 to 0.8 birth per woman in 2020 [20]. The combination of increasing life expectancy and decreasing fertility rates has contributed to a profound shift in the age structure of SK—the proportion of youth population over the past twenty years has declined significantly [21]. These important societal transformations could potentially result in shifts in the SK age-crime patterns through modifying socialization and social control experiences across generations.

It is plausible, therefore, that the difference between SK and US age-crime patterns, as observed in the Steffensmeier et al. study, was shaped by period and cohort effects and represents a unique pattern for the 2001–2010 timeline. To tease out these factors, researchers have to conduct an age-period-cohort (APC) analysis using age-crime data across time. Two studies published in 2023 sought to accomplish this by examining SK age-arrest data in the past forty years [22, 23]. Focusing on broad groupings of *violent offenses* (e.g., homicide, assault, etc) and *property offenses* (e.g., theft, fraud, etc), the Kang & Hureau (KH) and You’s studies [22, 23] concluded that *first*, their findings overall are consistent with Steffensmeier et al.’s cross-sectional analysis showing age-crime patterns divergent from HG invariance norm, and *second*, some shifts in the age-crime relationship have occurred which they interpret as possibly brought on by socioeconomic and demographic changes across the study period.

Both KH’s [22] and You’s [23] studies represent a thoughtful effort to address the influence of period and cohort effects on SK’s age-crime relationship. Caution is warranted, however, when interpreting these studies because their analyses did not delineate ordinary property crime (i.e. theft, robbery) as distinct offense types as was done in the Steffensmeier et al. study. Instead, they collapsed arrests for ordinary property crime (e.g., theft) into a summary property offense including embezzlement, breach of trust, possession of stolen goods, and fraud, which together account for a sizable portion of all property crime arrests in SK. This limitation is particularly noteworthy because the Steffensmeier et al. study found that the age curve for fraud is left-skewed and displays an older peak (mid-forties) whereas the age curve for ordinary property crime is right-skewed and displays an early peak (mid-late teens). Notably, while the age pattern for fraud diverged sharply from the HG invariance norm, the age pattern for ordinary theft matched well with the invariance norm and the prototypical US age-arrest curve.

An important added limitation of the KH study is that they relied on the APC ANOVA modeling method. Specifically, while the study estimated overall contributions of age, period, and cohort effects on the outcome, it did not provide estimations of age, period, and cohort coefficients for predicting the average age-crime curve while adjusting for period and cohort effects. Second, the APC ANOVA method focuses on recovering the independent and additive effects of age, period, and cohort, but it does not recognize the interdependence among these factors. As suggested in prior literature, period effects leading to social change may have a greater impact on some age (cohort) groups than others, modifying the age-crime curves [24–26]. The cohort effects resulting from these age and period interactions are not fully captured by the traditional APC ANOVA model.

The overall goal of the current study is to address the aforementioned shortcomings of the prior studies by analyzing age-specific arrest statistics for homicide, assault, fraud, and theft from SK covering the 1980–2019 timeline with the APC-I model developed by Luo and Hodges [25]. Specifically, I focus on two prominent questions that have not been evaluated in prior research: (1) *Are the average age-crime patterns in SK divergent from the HG norm after controlling for period and cohort effects*? and (2) *How do period and cohort effects modify SK age-crime patterns during the study period*? The APC-I model is well-suited for examining these research questions. As elaborated in the method and result sections, by treating cohort effects as age-by-period interactions, the APC-I model—*first*, is immune to the APC identification problem; and, *second*, it recognizes the interdependence between age, period, and cohort effects [25, 27]. In the following sections, I will first introduce the data and methods, followed by presenting the results, and then summarize how the findings help achieve the aims of this study and discuss their implications.

## Data and methods

This study compiled official age-arrest data with age-specific population data in South Korea from 1980 to 2019. The age-specific arrest statistics are published annually by the Supreme Public Prosecutor’s Office (SPO thereafter), which can be assessed through the government’s official statistics web portal. Details of this data and the justifications for using arrest data to measure age-crime patterns are available in the Data Appendix (S1 A in S1 Appendix). In the SPO data, age is coded as individual ages from 14–25, in five-year groupings for ages 26–30, 31–35, 36–40, and in ten-year groupings for ages 41–50 and 51–60 (age 41–50 is separated into 41–45 and 46–50 after 2014).

**Fig 1:**
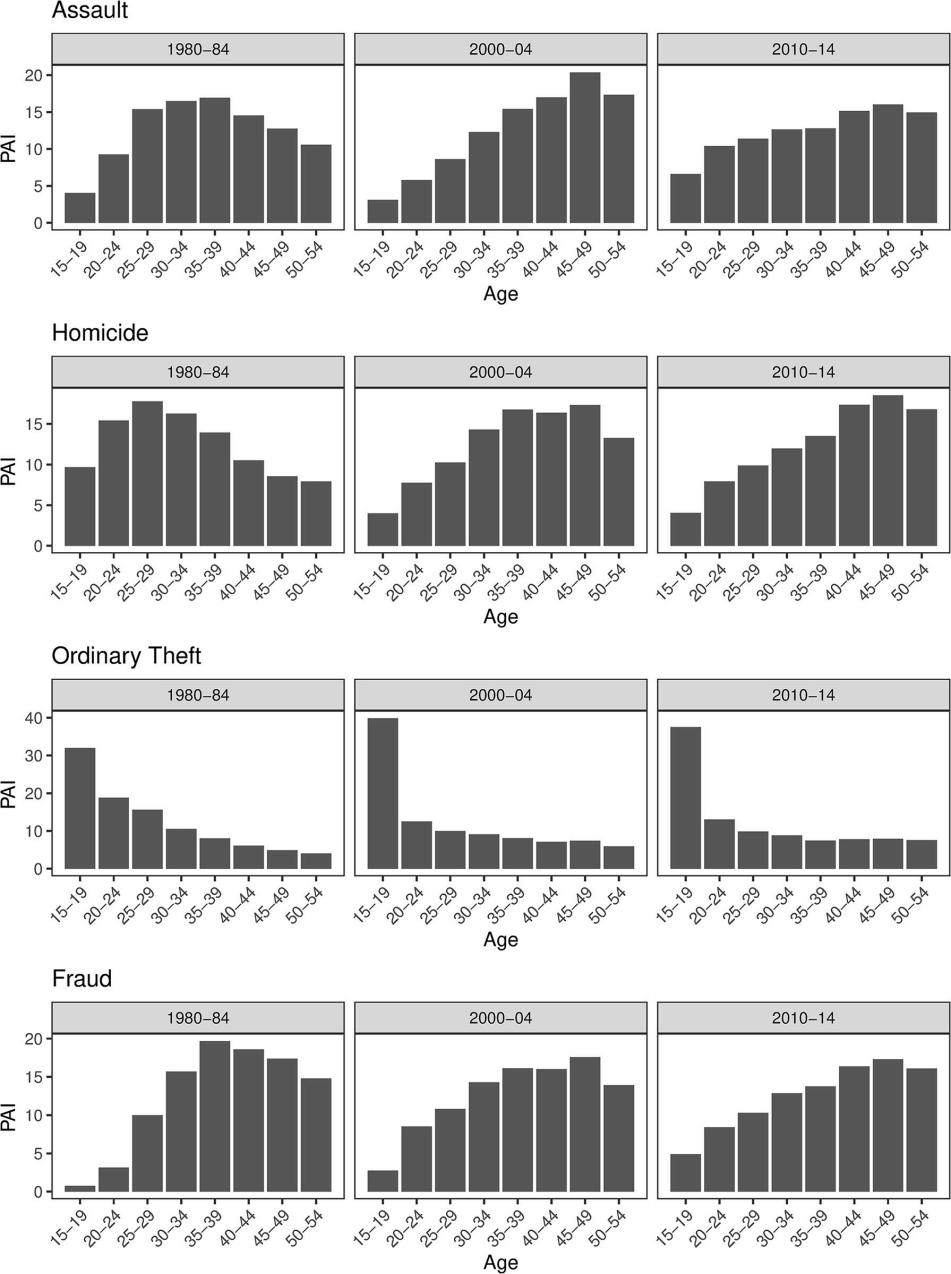
Age-arrest distributions by type of offense across historical periods in South Korea: Percentage age involvement (PAI). Notes: For parsimony, the analysis combines assault, battery, and willful infliction of bodily injury into the assault category as these three offenses share similar age-crime patterns across time and overlap in offense conduct. Ordinary theft combines theft with robbery offense for the same reason.

To create age categories with equal intervals for the analysis, I first apply linear interpolation to estimate arrest counts for single ages (ages 15, 16…53, 54). Details of the linear interpolation method are included in the Data Appendix (S1 A in S1 Appendix). We also conduct sensitivity analysis to examine if alternative interpolation methods could affect the age-crime patterns. Results are almost identical to our current findings and they are presented in the Data Appendix (S1 Fig 1 in S1 Appendix). All the single-year arrest counts from age 15 to 54 are collapsed into eight five-year age groups (i.e. age 15–19, 20–24, 25–29…50–54). The annual arrest data from 1980 to 2019 are averaged into eight five-year groupings (i.e. 1980–84, 1985–89…2015–2019) to reduce instability due to random fluctuations. This approach also could provide a better measure of potential cohort effects—for instance, a 1-year bulge in the population can be easily absorbed by different social institutions (i.e. labor market, schools), but a 5- or 10-year bulge cannot, which contributes to the cohort size effects on the labor market [11].

Age-specific arrest rates were then calculated using age-specific population data covering the 1980–2019 period obtained through the Korean Statistical Information Service portal (KOSIS). This paper focuses on four measures that are consistently recorded in the SPO data across the study period, including assault (summary of assault, battery, and willful infliction of bodily injury), homicide, ordinary theft (summary of theft and robbery), and fraud. For parsimony, the analysis combines assault, battery, and willful infliction of bodily injury into the assault category, and combines theft and robbery into the ordinary theft category, as these offenses share similar age-crime patterns across time and they overlap in offense conduct [8].

A combination of statistical procedures is used to assess the impact of period and cohort effects on SK age-crime patterns. The first part is a series of descriptive analyses, which examine the shape of the SK age-crime patterns across periods by data visualization and by comparing the key parameters of the distributions (i.e. peak age, ½ descending peak, skewness). For the descriptive analysis, the percentage age involvement (PAI) for each period is calculated using the age-specific arrest rates. The formula for calculating the PAI is:

$${PAI}_{ij}=\frac{r_{ij}}{\sum r_{ij}}*100,$$

where *r* = age-specific arrest rate, *i* = age category, and *j* = offense category. PAI is used to plot the age-crime curves and calculate summary measures of the age-crime distribution (e.g., peak age, one-half peak descending, skewness). PAI is defined as the percentage of arrests accounted for by each age group of interest (See details in Steffensmeier et al. 2017; 2019; 2020). It standardizes the rates as relative percentages so varying levels of crime rates in different periods are comparable to each other. The second part of the analysis involves modeling the age-specific arrest data with the age-period-cohort-interaction (APC-I) method developed by Luo and Hodges [25]. Details of the APC-I method are elaborated below after the descriptive analysis and along with the modeling results.

## Descriptive analysis

The age arrest distributions across periods (1980–84, 2000–04, 2010–14) for different offense types are presented in Fig 1. Three important patterns emerged from these plots. First, except for ordinary theft, the age-arrest distributions in SK diverge considerably from the HG invariant thesis that projects an adolescent-spiked right-skewed distribution. SK age-crime curves are more spread out with much older peaks as compared to the HG invariant thesis and arrest patterns observed in the U.S. [1, 6]. High arrest rates are consistently observed among middle age groups and have prevailed into the 50s in recent periods. Adolescents account for a very small proportion of arrests (with ordinary theft being the exception). Second, the shapes of age-crime curves have shifted somewhat toward older peak ages in the most recent decade. Third, the age distribution of ordinary theft is the only offense type that consistently displays an age pattern consonant with the HG projection.

Table 1 presents the key parameters of each age-arrest plot in Fig 1. The peak age indicates the age group that accounts for the highest proportion of arrests. For assault, homicide, and fraud, the peak ages are consistently beyond the adolescent age group, and they also have shifted from middle to older age groups over time. The half-peak descending shows the pace of desistance by indicating the age in the downslope at which the peak rate has decreased by half. Across assault, homicide, and fraud, most of the half-peaks are 55+, meaning that the half-peak descending is not reached within the age range in the current study. These results indicate that age-arrest patterns in SK typically peak at older ages and display a slower post-peak decline as compared with the HG invariance norm and with what generally is found in the U.S.

**Table 1:** Timing of crime by type of offense across periods.

	Peak			½ Peak Descending			Skewness^1^		
Offenses	1980–84	2000–04	2010–14	1980–84	2000–04	2010–14	1980–84	2000–04	2010–14
**Assault**	35–39	45–49	45–49	55+	55+	55+	-0.98	-0.30	-0.73
**Homicide**	25–29	45–49	45–49	45–49	55+	55+	0.11	-0.67	-0.36
**Ordinary theft**	15–19	15–19	15–19	25–29	20–24	20–24	1.14	2.13	2.13
**Fraud**	35–39	45–49	45–49	55+	55+	55+	-0.70	-0.97	-0.56

Notes: For parsimony, the analysis combines assault, battery, and willful infliction of bodily injury into the assault category as these three offenses share similar age-crime patterns across time and overlap in offense conduct. Ordinary theft combines theft with robbery offense for the same reason.

^1^A positive value (larger than 0.5) indicates a right-tailed distribution as predicted by the HG invariant thesis, a negative value (smaller than -0.5) indicates a left-tailed distribution and a value of around zero (between -0.5 and 0.5) reflects a symmetrical distribution (see also footnote 2). A rule of thumb for interpreting skewness of population data is (1) if skewness is less than -1 or greater than +1, the distribution is highly skewed; (2) if skewness is between -1 and -0.5, or between 0.5 and 1, the distribution is moderately skewed; (3) if skewness is between -0.5 and 0.5, the distribution is approximately symmetric [28]. It is important to note that if sample data is used (instead of population data), the interpretation of skewness depends on the sample size.

The last parameter presented in Table 1 is skewness, which measures whether a distribution is symmetrical, left-skewed, or right-skewed. A positive value (larger than 0.5) indicates a right-tailed distribution as predicted by the HG invariant thesis, a negative value (smaller than -0.5) indicates a left-tailed distribution and a value around zero (between -0.5 and 0.5) reflects a symmetrical distribution [28]. Except for ordinary theft, all skewness values are either negative or very close to zero (within -1 and 1), indicating that the distributions are nearly symmetrical and slightly left-skewed, which diverge from the strongly right-skewed age-crime patterns projected by the HG thesis.

As compared to the other offense types, however, the age curves for ordinary theft in SK closely resemble the HG invariant projection. The age-theft distributions have young peak ages at 15–19, with the half-peak descending reaching the early or late 20s, and display positive skewness values larger than one. Across the three periods, the distributions are consistently right-skewed with steep adolescent peaks and sharp post-peak decline. Some possible explanations for the atypical patterns of ordinary theft are discussed in the latter section.

In sum, the results of descriptive analysis support Steffensmeier et al.’s [8] conclusion that most offenses in SK have older and more spread-out age-arrest distribution than the HG invariance projection, with ordinary theft being the exception. This is also reported in Steffensmeier et al.’s study [8]. However, these results also reveal some changes in the age-crime curves across time that may reflect possible period and cohort effects. Next, I conduct the age-period-cohort analysis to examine this plausibility.

## Age-period-cohort analysis

Modeling age, period, and cohort effects simultaneously has been challenging because of their linear dependency: information about age is completely determined by period and cohort membership (i.e. age = period-cohort). A conventional model including these three factors is not identifiable because there is an infinite number of solutions that provide an identical fit to the data [29]. Prior APC methods apply specific constraints in the model (e.g. quality constraints, intrinsic estimator) [30, 31], but these constraints involve empirical research assumptions that are difficult to verify [32, 33]. Moreover, conventional APC approaches focus on teasing out the independent and additive effects of age, period, and cohort but overlook the interdependence of the three factors. In doing so, regardless of the identification strategies, traditional APC models implicitly assume that cohort effects can occur independently from social changes (i.e. period effects), which departs from Ryder’s seminal work [16] that defines cohort effects as age-period-specific. The APC-I model addresses these issues (see methodological details in [25, 27]). Instead of treating age, period, and cohort factors as additive and independent, the APC-I approach considers the interdependence of the three factors by modeling cohort effects as age-by-period interactions. The APC-I model is specified as a generalized linear model below:

$${g(E(Y}_{ij}))=\mu +\alpha_{i}+\beta_{j}+{\alpha \beta }_{ij(k)},$$ (1)

where *g*(*E*(*Y*_*ij*_)) denotes the link function of the expected arrest counts *Y* for the *i*th age group in the *j*th period; *μ* denotes the global mean of the observed arrests; *α*_*i*_ denotes the main age effects associated with the *i*th age category; *β*_*j*_ denotes the main period effects associated with the *j*th period; *αβ*_*ij*(*k*)_ denotes the interaction of the *i*th age group and *j*th period group, which corresponds to the effect of the *k*th cohort. The age-by-period interaction terms *αβ*_*ij*(*k*)_ in Eq (1) are used to compute inter-cohort deviation and intra-cohort life course dynamics. Given the interactive nature of the cohort effects, as modeled in the APC-I framework, the inter-cohort effects should be interpreted as whether a cohort has a higher or lower arrest rate than the predicted arrest rate determined by age and period main effects only. A methodological appendix of a full APC-I analysis, including the interpretations and the strengths of the model, is presented in the Technical Appendix (S1 B in S1 Appendix). Given that the primary objective of the current study is to estimate age-crime distributions in SK while accounting for the period and cohort factors, the main analysis focuses on the age main effects (*α*_*i*_) in Eq (1). Additional modeling statistics are included in the technical appendix (S1 C in S1 Appendix).

**Fig 2:**
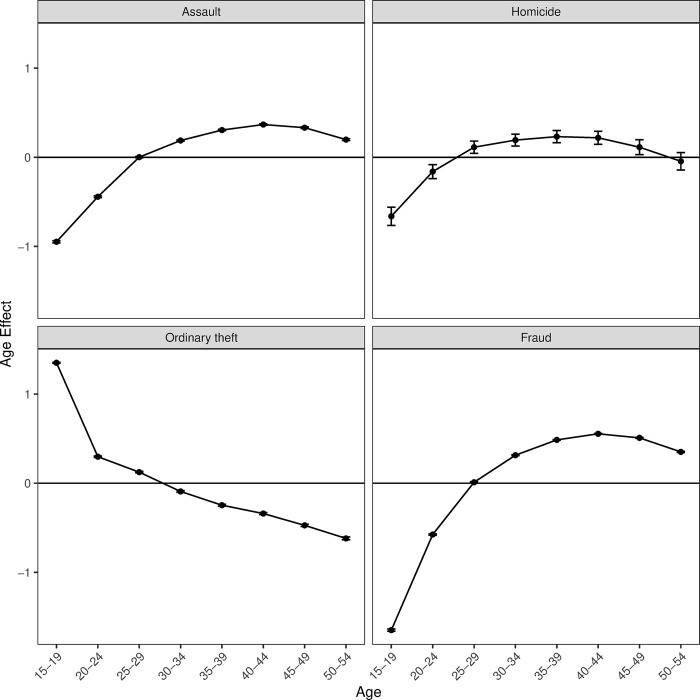
Estimated age main effects on arrest rates of different offense types, controlling for period and cohort effects. Notes: Figures are estimated main age effects on different offenses when period and cohort effects are controlled. The horizontal solid line represents zero deviation from the global mean (intercept of the APC-I model). Points above the zero line represent positive deviations from the global mean, suggesting higher-than-expected risks of arrest; points below the zero line represent negative deviations from the global mean, suggesting lower-than-expected risks of arrest. The 95% confidence intervals are also presented for each point estimate. However, because the standard errors for the age effects of assault, ordinary theft, and fraud are very small, the error bars in these plots overlap with the dots.

Poisson regression is used to generate estimates for count data and the age-period-specific population is included as an *offset* term in the equation to account for the variation of the population at risk in different periods. This approach is essentially the same as modeling crime rates but it allows researchers to model rates without violating assumptions of the regression model. This study opted for the Poisson model rather than the negative binomial model because of the limited degrees of freedom inherent in the aggregate-level age-crime data. Detailed justifications for the model choice are included in the Methodology Appendix (S1 B and S1 Fig 2 in S1 Appendix). Table 2 demonstrates the data structure for an APC analysis using homicide as an example. For each offense type, there are 8 five-year age categories (i.e. age 15–19, 20–24, …50–54) and 8 five-year period categories (i.e. 1980–84, 1980–84, …2015–19), which results in 15 five-year birth cohorts (i.e. 1930–34… 2000–04) in the data. There are 64 observations (age-period-specific counts) for each offense type.

**Table 2:** Homicide arrest rates by age, period, and cohort.

	Arrest rate	Period							
Age	*(Cohort)*	1980–84	1985–89	1990–94	1995–99	2000–04	2005–09	2010–14	2015–19
15–19	Arrest rate	2.0	2.4	2.3	1.4	0.9	0.6	0.9	0.8
	*(Cohort)*	*(c1965)*	*(c1970)*	*(c1975)*	*(c1980)*	*(c1985)*	*(c1990)*	*(c1995)*	*(c2000)*
20–24	Arrest rate	3.2	3.0	3.1	2.1	1.8	1.2	1.7	1.5
	*(Cohort)*	*(c1960)*	*(c1965)*	*(c1970)*	*(c1975)*	*(c1980)*	*(c1985)*	*(c1990)*	*(c1995)*
25–29	Arrest rate	3.7	3.7	3.4	3.2	2.4	2.0	2.1	2.0
	*(Cohort)*	*(c1955)*	*(c1960)*	*(c1965)*	*(c1970)*	*(c1975)*	*(c1980)*	*(c1985)*	*(c1990)*
30–34	Arrest rate	3.4	3.5	3.3	3.6	3.3	2.3	2.6	2.2
	*(Cohort)*	*(c1950)*	*(c1955)*	*(c1960)*	*(c1965)*	*(c1970)*	*(c1975)*	*(c1980)*	*(c1985)*
35–39	Arrest rate	2.9	2.9	3.2	3.6	3.9	3.2	2.9	2.2
	*(Cohort)*	*(c1945)*	*(c1950)*	*(c1955)*	*(c1960)*	*(c1965)*	*(c1970)*	*(c1975)*	*(c1980)*
40–44	Arrest rate	2.2	2.3	2.8	3.6	3.8	3.7	3.7	2.7
	*(Cohort)*	*(c1940)*	*(c1945)*	*(c1950)*	*(c1955)*	*(c1960)*	*(c1965)*	*(c1970)*	*(c1975)*
45–49	Arrest rate	1.8	1.5	2.2	2.9	4.0	3.7	4.0	3.1
	*(Cohort)*	*(c1935)*	*(c1940)*	*(c1945)*	*(c1950)*	*(c1955)*	*(c1960)*	*(c1965)*	*(c1970)*
50–54	Arrest rate	1.7	1.2	1.5	2.2	3.1	3.5	3.6	3.2
	*(Cohort)*	*(c1930)*	*(c1935)*	*(c1940)*	*(c1945)*	*(c1950)*	*(c1955)*	*(c1960)*	*(c1965)*

Note: Each diagonal of the table represents homicide arrest rates per 100,000 of a specific cohort across different ages. For example, homicide arrest counts for Cohort 1965 were 2.0 at age 15–19 (in 1980–84), 3.0 at age 20–24 (in 1985–89), and 3.2 at age 50–54 (in 2015–19).

**Fig 3:**
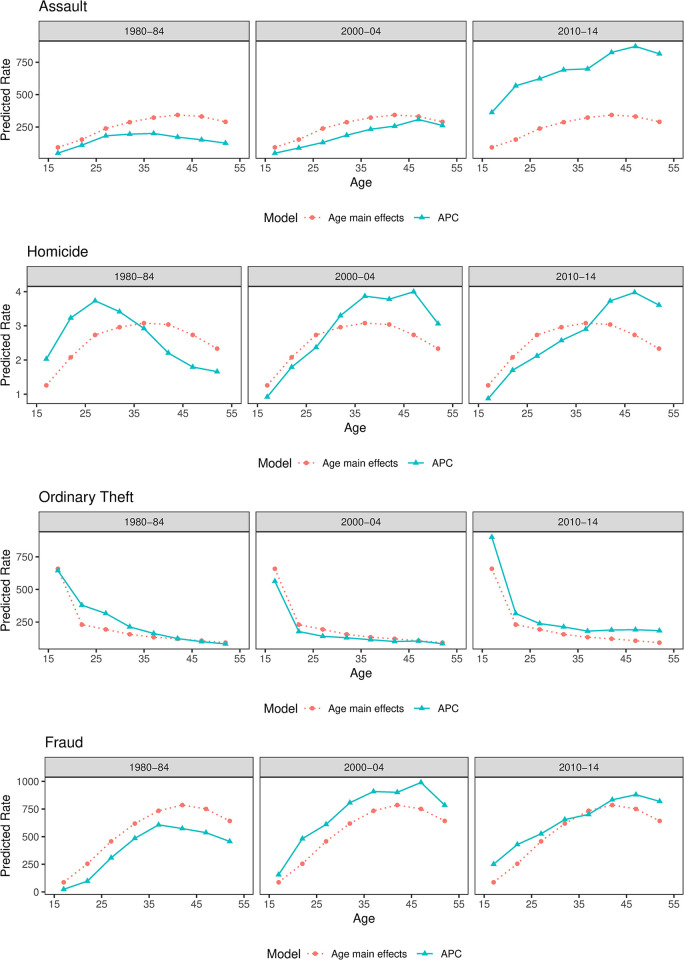
Predicted age-crime distributions by offense types and models across historical periods. Notes: The red dotted curve in each plot represents the predicted age curve with age main effects only, controlling for period and cohort effects, which we also define as the average time-invariant curve. The blue solid curve represents the predicted age curve of the APC model (i.e., with age, period, and cohort effects). The deviation of the blue solid curve from the red dotted curve indicates how period and cohort effects modify the average age-arrest curve across different periods.

In the APC analysis, I estimate the age, period main effects, and age-by-period interaction terms for each offense type by fitting four different APC-I models. Because the focus of this paper is on age effects, I first present the estimated main age effects for each offense type (Fig 2) and then visualize how the period and cohort effects together modify the age-arrest curves in different periods (Fig 3). Detailed information on the full APC-I models for each offense is included in the supplemental materials (S1 C in S1 Appendix). S1 Table 1 in S1 Appendix includes model statistics for age and period effects, and S1 Table 2 in S1 Appendix presents estimates for inter-cohort differences. S1 Fig 3 in S1 Appendix visualizes period main effects and inter-cohort differences based on the APC-I models.

### Average age-arrest patterns in South Korea

In each APC-I model, the age coefficients represent the main effects of being in a specific age group as compared to the global arrest means (the intercept of the model), holding period, and cohort effects constant. For parsimony, I plot the age main effects of each offense in Fig 2, which represents the average age-arrest distribution after controlling the influence of period and cohort. The horizontal line in each plot represents zero deviation from the mean (i.e., the intercept). A positive deviation from the horizontal line indicates higher-than-average arrest rates, while a negative deviation indicates the opposite. For example, the estimated age coefficients for assault in SK at ages 15–19 and 20–24 are -.949 and -.443, which means that location in these two age groups reduces the risk of arrest for assault by 61% (e^- 0.949^–1) and 36% (e^- 0.443^–1) relative to the mean (See S1 Table 1 in S1 Appendix). The estimated age coefficient for assault at age 40–44 is .367, which means age 40–44 has a 44% higher chance of being arrested for assault than the mean. As depicted in Fig 2, ages 40–44 have higher risks for assault arrests than adolescents (age 15–19) and young adults (age 20–24).

Except for ordinary theft, the average age-arrest patterns in SK depart significantly from the HG invariant projection. When period and cohort effects are held constant, the average age curves for assault, homicide, and fraud are spread out and slightly left-skewed, with the highest crime rate observed among people in their 30s and 40s. Adolescents (age 15–19) display lower arrest rates than the oldest age group (i.e. age 50–54), which contrasts sharply with the HG invariant thesis and age-crime patterns in some Western countries. These results provide additional support for the conclusions of prior literature, indicating that, even after controlling for period and cohort effects, crime in SK is more concentrated among middle-age groups than young age groups (except for ordinary theft).

### Period and cohort effects on the age-arrest curves

After estimating the average age-crime pattern, I next examine the second research question—how period and cohort effects have modified the average age-arrest patterns. Fig 3 compares two curves in each offense-period-specific plot. The red dotted line represents the average age-arrest curve after controlling for period and cohort effects, which replicates the pattern shown in Fig 2. The blue solid line represents the predicted age-arrest curves when the estimated age, period, and cohort effects are included. The discrepancy between the average age curve (red dotted line) and the APC-I curve (blue solid line) indicates how period and cohort effects modify age-specific arrest rates in each of the three periods. For parsimony, I select the periods of 1980–84, 2000–04, and 2010–14 for comparison, but the plots for other periods are available upon request.

Before discussing the results of Fig 3, I first clarify the difference between period and cohort effects regarding their influence on the age curves. Because period main effects are universal for all age groups, when period effects *only* are present (i.e., small, or negligible cohort effects), the age-arrest distribution displays approximately the same shape as the average age-arrest curve. For example, the shape of the age-fraud distribution in 1980–84 (blue solid line in the last panel of Fig 3) matches the average time-invariant age-fraud curve (red dotted line in the last panel of Fig 3) with lower arrest rates across all age groups than the red curve. This pattern indicates that period effects have contributed to lower fraud rates for all ages in 1980 and that there are trivial cohort effects, resulting in little or no change in the overall shape of the age-fraud curve. When there are strong cohort effects present, however, the shape of an age curve may shift significantly. For instance, cohort effects have contributed to an older peak (ages 45–49) observed from the homicide APC curve in 2010–14 as compared to the average age-homicide curve (ages 35–39). The birth cohort reaching their late 40s in 2010–14 has higher-than-expected homicide rates, while the young adults and middle-aged cohorts in 2010–14 have lower-than-expected theft rates, which together have modified the age-homicide curve from a symmetrical distribution to a left-skewed distribution.

Fig 3 reveals both consistency and some change in age-arrest patterns across time. Period and cohort effects have modified the shapes of age-homicide and age-fraud patterns across time, but the shapes of assault and theft distributions have remained relatively stable. Period effects have contributed to a substantial increase in the level of assault arrests, but the age-assault curves are consistently spread out across the three periods. For ordinary theft, the distribution with a high adolescent peak and sharp decline after the peak are consistently found across the three periods, with only small changes yielded after considering cohort and period effects. Age-homicide curves display the most substantial changes across time as compared to the other three offenses. It shifts from peaking in the late 20s (in 1980–84) to a much older peak in the late 40s (in 2010–14). Cohort effects also have shifted the peak age of fraud to older age groups in 2010–14. In sum, period and cohort effects have shifted the age-crime curves in SK to a certain extent, but the effect size varies across offense types.

## Discussion

The current study extends Steffensmeier et al.’s [8] cross-sectional analysis of SK age-arrest patterns by analyzing the longitudinal age-specific crime data from 1980 to 2019 with APC analysis. Three important findings emerged from the analyses and their implications will be discussed below. First, the most notable observation is the consistent divergence from the HG norms in the shape of assault, homicide, and fraud distributions, which supports the conclusion of Steffensmeier et al. ‘s [8] study. The average age-crime patterns of these three offenses are robustly spread out with late peaks between 30 and 50 years old, even after controlling for period and cohort effects. These findings align with the neo-modernization approach and assert that the broad cultural heritage of a society “leaves an imprint on values that endures” notwithstanding economic and social development [34]. Despite the dramatic social and demographic changes in SK over the past four decades, the collectivist cultural values and the age hierarchy in SK continue to exert profound and persistent impacts on the age-graded norms in SK. These norms imply stringent social control and monitoring of adolescents but relatively weak control over middle age groups, resulting in relatively low youth crime and spread-out age-crime distribution across time [8, 22].

The second important finding is the anomalous age-crime patterns for ordinary theft, which differ significantly from other offenses in SK and demonstrate overall consistency with the HG invariant projection. Even after controlling for period and cohort effects, the age-theft curve still yields steep adolescent peaks followed by sharp post-peak declines. This pattern was also discussed in Steffensmeier et al. ‘s analysis [8] but with cross-sectional age-theft data from the 2000s. Combining longitudinal SK age-arrest data with advanced APC-I modeling methods, this study provides additional evidence that the inverted-J shape age-theft pattern is not due to period or cohort effects.

Two possible explanations to account for the age-theft pattern can be drawn from extant writing on youth offending and prior research in SK. First, arrests for ordinary theft among SK adolescents typically involve minor law violations, such as shoplifting small items from shopping malls or stealing items such as cell phones and bicycles. This age pattern is consistent with Steffensmeier et al.’s argument that teenagers are more likely to engage in low-yield delinquencies, such as theft, because they require relatively less sophisticated skills but still yield small financial gains and enhance peer group status [6]. Second, adolescents involved in ordinary theft tend to be marginalized youth such as those who have dropped out of school, run away from home, or been isolated from their families because of poor academic performance [8, 35]. It is argued that these marginalized adolescents are at higher risk of hanging out with delinquent groups and being exposed to messages and situations conducive to low-yield property crimes [14]. Furthermore, it is also plausible that the adolescent peak for theft in SK partly reflects greater law enforcement surveillance, which seems to particularly target marginalized adolescents in disadvantaged neighborhoods, leading to high youth arrest rates [36].

Third, the results also suggest that cohort effects have modified somewhat the SK age-arrest curve. Cohorts in the late 40s and early 50s in 2010–14 have higher than expected arrest rates for homicide and fraud, shifting the age-crime curves from symmetrical in 1980–84 (i.e., assault) or slightly right-skewed (i.e., homicide) to strongly left-skewed in 2010–14. These cohorts involve the late baby boomer generation in SK born in early 1960. While beyond the scope of this paper to address, future research should investigate whether or not specific cohort mechanisms such as cohort size [11], economic development, or other unique experiences of the baby boomer cohorts help to account for the small-to-moderate-sized cohort effects on SK age-crime patterns.

### Limitations

Notwithstanding the noteworthy findings emphasized above, this research has limitations. The reliance on official police data published by law enforcement agencies raises the possibility that age-arrest patterns across the study period have been affected by changes in policing or other formal social control practices. The study addressed this important limitation of the data with the APC analysis as well as a series of robustness checks. First, the APC analysis utilized in the study can effectively control for a substantial portion of changes in policing as well as other policy changes. If there are changes in policing or other policies that affect crime across all ages in the population in a specific period (e.g. the shifts from colonial policing to democratic policing in the Grand Reform since 1999), these changes are captured and controlled by the period main effects; if there are changes in policing or other policies that affect crime in specific age groups during a specific period (e.g. the reform of the Juvenile Act), these changes are controlled by the cohort effects (age-by-period interactions). Thus, the average age-crime patterns presented in Fig 2 have taken into account changes in law enforcement policies to a certain extent by controlling for period and cohort effects.

Second, to address the concern that the different age-crime patterns observed in SK may be a result of more lenient practices towards adolescents, I conducted a supplemental analysis (S1 D in S1 Appendix) with the post-adolescence age-arrest data in SK (i.e. ages 20 and above). Considering that discretionary policing of teen law violators likely ends around age 19 or shortly after [8, 37], a finding showing no divergence between the SK post-adolescence age-crime distributions and the HG age-crime invariance thesis would suggest that law enforcement or reporting discretion might be in play. However, as shown in the Technical Appendix (S1 Fig 4 in S1 Appendix), except for theft, all average age-crime patterns after controlling for period and cohort effects remain skewed to the left and the age-specific crime rates continue to rise well past young adult ages. It provides additional evidence affirming that the divergence of SK age-crime data is not merely a consequence of less strict law enforcement toward teen law violators.

Third, the adolescent-spiked age curve observed for ordinary theft also serves as compelling evidence against the alternative notion that the divergence in age-crime patterns is a result of law enforcement discretions. If the adolescent spike in arrests for theft is seen as real, it is unlikely that stringent enforcement is selectively applied only to youth involved in theft while simultaneously maintaining lenient policing toward other forms of adolescent law violation [8]. Furthermore, the older average age-crime schedule observed for homicide, a crime widely regarded as the most reliably reported crime with a lower likelihood of being affected by policing variations across ages [38], adds further confidence that the dissimilar age-arrest patterns in SK are not solely a result of differential policing. Lastly, qualitative data gathered via interviews with SK police officers and criminologists in prior studies indicate that the spread-out age-crime distributions in SK indeed reflect significant differences in the cultural and social contexts of SK that influence individuals’ criminal behaviors [8].

Therefore, while recognizing the limitations of arrest data and the impacts of changes in law enforcement policies and practices, the current study identified additional evidence with supplemental analysis to mitigate potential biases associated with these limitations and to ensure the robustness of the findings.

## Conclusions

The current age-period-cohort analysis of SK data from 1980 to 2019 confirms that age-crime patterns in SK robustly diverge from the HG invariant thesis and prototypical age-arrest patterns in the U.S., except for ordinary theft which displays the inverted J-shaped curve projected by HG. Criminal offending in SK, overall, is more heavily concentrated among midlife age groups (older than 30), whereas teens or young adult age groups account for a relatively small proportion. These results provide additional support to prior cross-national research challenging the HG invariance thesis, questioning the developmental-neurobiological approaches that attribute the age effects on crime to preprogrammed neurobiological changes. The current findings imply that, despite the potential involvement of biological factors in the developmental process from adolescence to adulthood, the age-crime schedule is also strongly shaped by country-specific processes [3, 8].

More broadly, the current study underscores the importance of utilizing international data and adopting a cross-cultural comparative perspective. Non-Western studies on crime are essential for providing a reevaluation of the theories of crime as well as other human behaviors developed predominantly based on data from Western countries [39]. The evidence of the current study, coupled with the recent empirical studies showing divergent age-crime patterns in other Asian countries [3, 7, 40], highlights that cultural and historical context matters in shaping human behaviors. A scholar with knowledge of the age-crime patterns in South Korea might formulate different theoretical hypotheses as compared to those scholars focused on the U.S. or other major Western developed countries. Moreover, these cross-national challenges may offer new insights and reflections for the development of criminal justice policies in Western countries, especially on how to effectively reduce youth crime.

Besides the theoretical and policy contributions, the study also provides an important methodological toolkit for future studies seeking to disentangle period and cohort effects on the age-crime patterns across time. The APC-I model applied in this study provides an innovative and robust methodology for estimating average age-crime patterns across time as well as for investigating how period and cohort effects might influence or modify the age curves. Future research aiming at examining the age-crime relationship or life course trajectories across time and/or across birth cohorts in different countries may consider adopting the current study as a template.

## Supporting information

S1 Appendix Technical appendix. (DOCX)
