# Supplementary material for: Examining the stability and change in age-crime relation in South Korea, 1980–2019: An age-period-cohort analysis
Source: PLoS One. 2024 Mar 29;19(3):e0299852. doi: 10.1371/journal.pone.0299852 (PMC10980243; doi:10.1371/journal.pone.0299852)
Supplement: S1 Appendix — (DOCX) [file pone.0299852.s001.docx]

# S1. Technical Appendix

# A. Data appendix

The replication files for this study, including the dataset and the R scripts, are openly available on GitHub at (<https://github.com/irisyunmeilu/SK_APC.git> ). This appendix provides technical details of the data and how the author addresses some limitations in the data.

## Data source & data cleaning

There are three major data sources for measuring criminal behaviors: official crime statistics (e.g. offense and arrest data from criminal justice agencies), victimization surveys (e.g. National Crime Victimization Surveys in the U.S.), and self-report data [1]. Arrest data stands out in SK as the sole source offering age-specific information spanning multiple decades and cohorts. Victimization surveys are valuable for addressing underreported crime—an important limitation of official crime statistics—but rely on victims' reporting and consequently lack accurate age information of the offender [2]. While self-report surveys (e.g. Add Health, NLSY in the U.S.) are considered excellent alternatives for age-specific offending information, to the best of the author’s knowledge, no self-report dataset in South Korea measures delinquency from adolescence across cohorts. Therefore, while acknowledging the limitations of arrest data, it remains the most suitable option for examining the age-crime relationship across time in SK. In addition, due to these relative advantages, arrest data has been extensively utilized in age-period-cohort analyses of crime in prior literature in the U.S. and other countries.[3–9].

The age-specific offender arrest data used in the current study is downloaded from the Analytical Statistics on Crime via the Crime and Criminal Justice Statistics data portal: <https://www.crimestats.or.kr/portal/stat/easyStatEngPage.do;jsessionid=Sx2vu5MPelzzlzr8YQBdejVQYipYpgZM6Dklh3A96mSbaWCwJ6qN8YlU21h4NS7Q.ccjs_web_servlet_engine1>. This data was compiled and updated annually by the Supreme Public Prosecutor’s Office (SPO). One limitation of the data is that the age-crime categories are not consistently recorded across years in the SPO data. For data recorded before 1994, the last age group is 61 and older. For data compiled after 1994, arrest information for ages 61-70 and age 71 and above are available. Because of this inconsistency, the study truncates the data and limits the analysis to ages 15 to 51-60.

## Interpolation and robustness check

Another limitation of the data is that the age categories are not coded in consistent intervals. For data collected before 2014, age is coded as individual ages from 14-25, in five-year groupings for ages 26-30, 31-35, 36-40, and in ten-year groupings for ages 41-50 and 51-60. For data collected in and after 2014, age is coded as individual ages from 14-25, in five-year groupings for ages 26-30, 31-35, 36-40, 41-45, 46-50, and in ten-year groupings for ages 51-60. To create consistent age categories for the analysis, I apply linear interpolation to estimate arrest counts for single ages (ages 15, 16…53, 54).

The linear interpolation method has been widely used in social sciences for estimating missing data and for estimating trends with inconsistent age or period intervals [10–13]. It assumes linear changes between two known data points; thus, it uses linear polynomials to construct new data points between each two known observations. For instance, for data before 2008, we first disaggregate the 10-year age groups of age 30-39, 40-49, and 50-59 using the linear interpolation function in R (“*approx*” function) and then rearrange the age group to 5-year increments. The same approach is for the 10-year age groups of age 40-49 and 50-59 in the data after 2008.

We opt for linear interpolation in the main analysis for its parsimony, but we also conduct a supplemental analysis with the cubic spline interpolation technique to ensure our results are consistent across different interpolation methods [14]. Cubic-spline interpolation is a special case for spline interpolation where a set of piecewise cubic functions are used to interpolate and smooth a set of data points. It is also a commonly used interpolation method in social science research [14–17]. S1 Fig1 below replicates Fig1 in the main analysis and demonstrates the histogram of the age-crime distribution of three periods in SK based on cubic-spline interpolation techniques (“*spline*” function in R with “natural” method). The interpolated results for single-year estimates vary slightly from the same estimates based on the linear interpolation results, but once we re-arrange the data into five-year age categories to reduce instability, the interpolated patterns are almost identical across the two interpolation techniques. The patterns depicted in S1 Fig1 (based on cubic spline interpolation) mirror those observed in Fig1 (based on linear interpolation). Replication of the age-period-cohort analysis also demonstrates consistent findings.

## S1 Fig 1. Age-arrest distributions by type of offense across historical periods in South Korea based on cubic spline interpolation method.

**
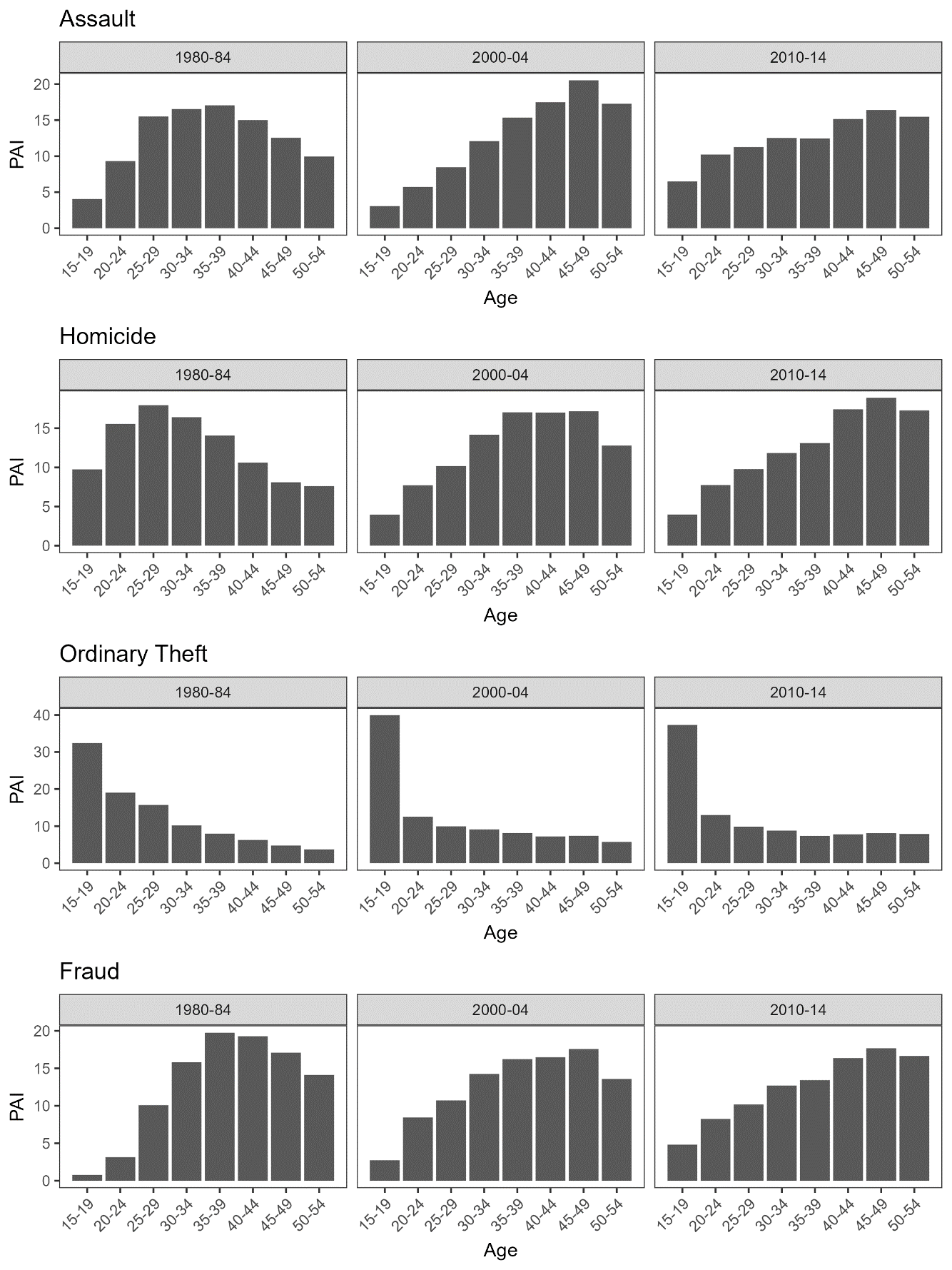
**

Notes: For parsimony, the analysis combines assault, battery, and willful infliction of bodily injury into the assault category as these three offenses share similar age-crime patterns across time and overlap in offense conduct. Ordinary theft combines theft with robbery offense for the same reason.

# B. Methodology appendix of the APC-I model

The APC-I model used in the current study conceptually differs from the methods developed under the traditional APC framework (e.g. Intrinsic Estimator (IE), the APC-Mixed model). The traditional methods attempt to estimate the independent and additive effects of age, period, and cohort on crime trends. In doing so, they implicitly assume that cohort effects can occur when the influence of social changes on the outcome is uniform across age groups. That is, regardless of the identification strategies, they all attempt to estimate the independent and additive effects of age, period, and cohort on crime trends. However, as argued in previous studies [3,18], this traditional framework contradicts the demographic literature that clearly defines cohort effects using an age-period specification [19,20]. Thus, to address the limitations in the traditional APC framework, the APC-I models cohort effects as a structure of the interaction terms between age and period main effects, considering cohort effects as differential period effects depending on age. By so doing, the APC-I model acknowledges and accommodates the inherent interdependence between age, period, and cohort.

Following the steps presented in Luo and Hodges[3,18], a full APC-I model that aims at quantifying the age main effects, period main effects, inter-cohort deviations, and the intra-cohort life course dynamics in aggregate level data should include three steps:

(1) A global deviance test. This test is conducted to examine whether there is variation in the outcome uniquely attributed to cohort membership by comparing the deviance score of a full APC-I model with a reduced model with age and period main effects only (${g(E(Y}_{ij}))=\mu+\alpha_{i}+\beta_{j}$). A significant test result indicates that the full model provides a better fit for the data than a partial model and cohort effects are present. All of the global deviance tests in the SK data for different offenses are statistically significant.

(2) Inter-cohort deviation. This involves estimating the average inter-cohort deviation and conducting a statistical test for each cohort. As shown in Table 2 of this paper, the age-by-period interaction effects in the same diagonal are corresponding to a specific cohort. Using the age-by-period interaction estimates (${\alpha\beta}_{ij(k)}$ ) in equation (1) of the manuscript) from each diagonal, we compute the average inter-cohort differences. A positive deviation indicates that this cohort has a higher arrest rate than the predicted arrest rate determined by age and period main effects only, whereas a negative deviation indicates the opposite. Z-tests are conducted to examine whether each of the inter-cohort deviations is statistically different from zero.

(3) Intra-cohort life course dynamics. The intro-cohort life course dynamics are examined by estimating the linear change in the interaction terms (${\alpha\beta}_{ij(k)}$ in equation (1) of the manuscript) of each cohort. Specifically, the linear orthogonal polynomial contrast of the corresponding age-by-period interaction terms will be calculated to determine whether the cohort’s average arrest risks accumulate, remain stable, or diminish across the life course.

Since the focus of the current paper is on the age effects, steps (2) and (3) presented above are not included in the main analysis of the manuscript but some of these results are presented in the supplemental materials (See S1 section C below). More details on how to calculate and interpret each step of the APC-I analysis can be found in Lu and Luo’s original study[3].

## The Sum-to-Zero coding scheme

All of the APC-I models are estimated using the sum-to-zero coding in R. Different from the conventional modeling approach that uses an age or period category as the reference group, the coefficients estimated using the sum-to-zero coding represent the deviation from the grand mean of all the observations. This approach also makes the interpretation of interaction terms easier as each coefficient of the interaction term represents the deviation from the expected values based on the main effects. Moreover, this approach also allows us to adjust for the arrest level differences across comparisons (e.g., different offense types)—that is, we can compare the estimated coefficients across offenses as the mean differences across offenses are held constant in the model.

## Justification for Using an APC-I Poisson Model

The current study opted for the Poisson model because of the limited degrees of freedom inherent in the aggregate-level age-crime data, prohibiting it from fitting a negative binomial (NB) model with the APC-I approach. In the SK aggregate-level age-crime data, each age-period-cohort-specification has only one observation (i.e. age-period-cohort-specific crime rate), resulting in limited degrees of freedom when simultaneously estimating age main effects, period main effects, and the age-by-period interactions in an APC-I model. While the negative binomial model can address the overdispersion issue in the crime data, the data’s insufficient degrees of freedom hinders the estimation of an APC-I NB model. In contrast, the Poisson model, which relies on one parameter (as compared to two parameters used in the NB model), provides one extra degree of freedom and enables the specification of an APC-I model with the aggregate crime data.

However, crime data often displays overdispersion, a situation where a Poisson model might underestimate the standard errors and overstate the significance of the regression parameters, consequently providing misleading inferences [21]. Therefore, a negative binomial model is often considered superior to a Poisson model in such cases. However, this concern is less pertinent to the current study, as it relies on population data rather than sample data. Since the primary research objective is to quantify age effects on crime in SK while accounting for period and cohort effects, the SEs and statistical significance are included only as a reference [22]. Interpretation of such models based on population data should focus on the effect sizes rather than the SEs and significance because they represent population parameters rather than sample statistics [23–25].

To further elaborate on this issue and address the concern of overdispersion, this study compared the age estimates of a Poisson model and a negative binomial model by fitting the data with an AP model— a partial model incorporating age and period main effects but does not control for age-by-period interactions. Estimating a negative binomial model is feasible in this case because a partial AP model requires fewer degrees of freedom than the full APC-I model. As depicted in the figure below (S1 Fig2), it is evident that while the plots with negative the binomial model exhibit larger standard errors for the estimates as compared to those of the Poisson model due to the overdispersion problem, the overall age-crime patterns, after controlling for period changes, are comparable across the two models.

In sum, while the study recognizes the limitations of using a Poisson model for crime data, these limitations are less of a concern to the findings of the current paper. This is attributed to the reliance on population data and the primary focus on quantifying the average age-crime patterns rather than emphasizing the significance of a specific age term. Most of the recent studies utilizing the APC-I framework to analyze aggregate-level crime data also apply the Poisson model in their analysis [3,9].

## S1 Fig 2. Comparing patterns of age effects on arrest rates based on a partial AP Poisson model and a partial AP Negative Binomial model.

1. Poisson Model b. Negative Binomial Model


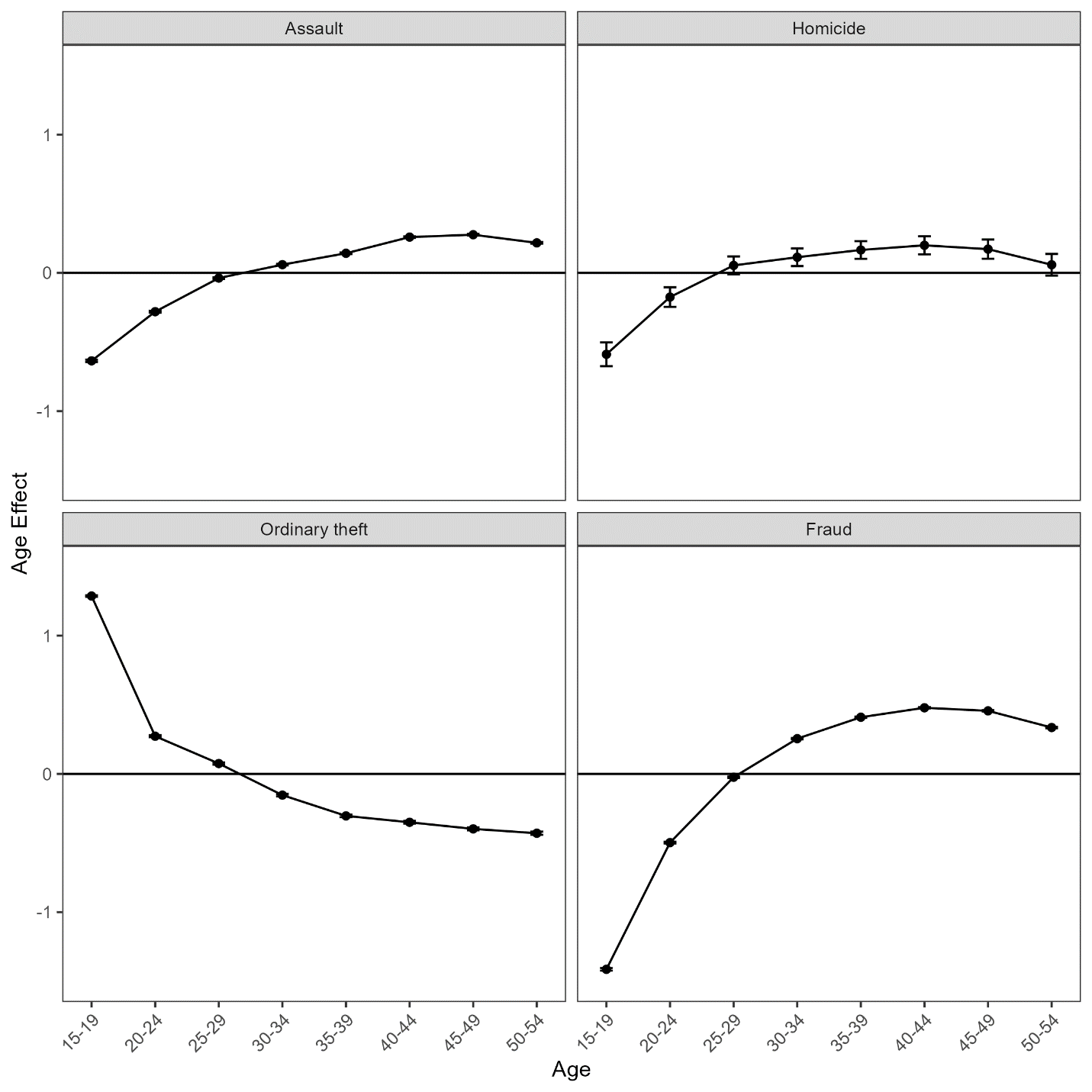

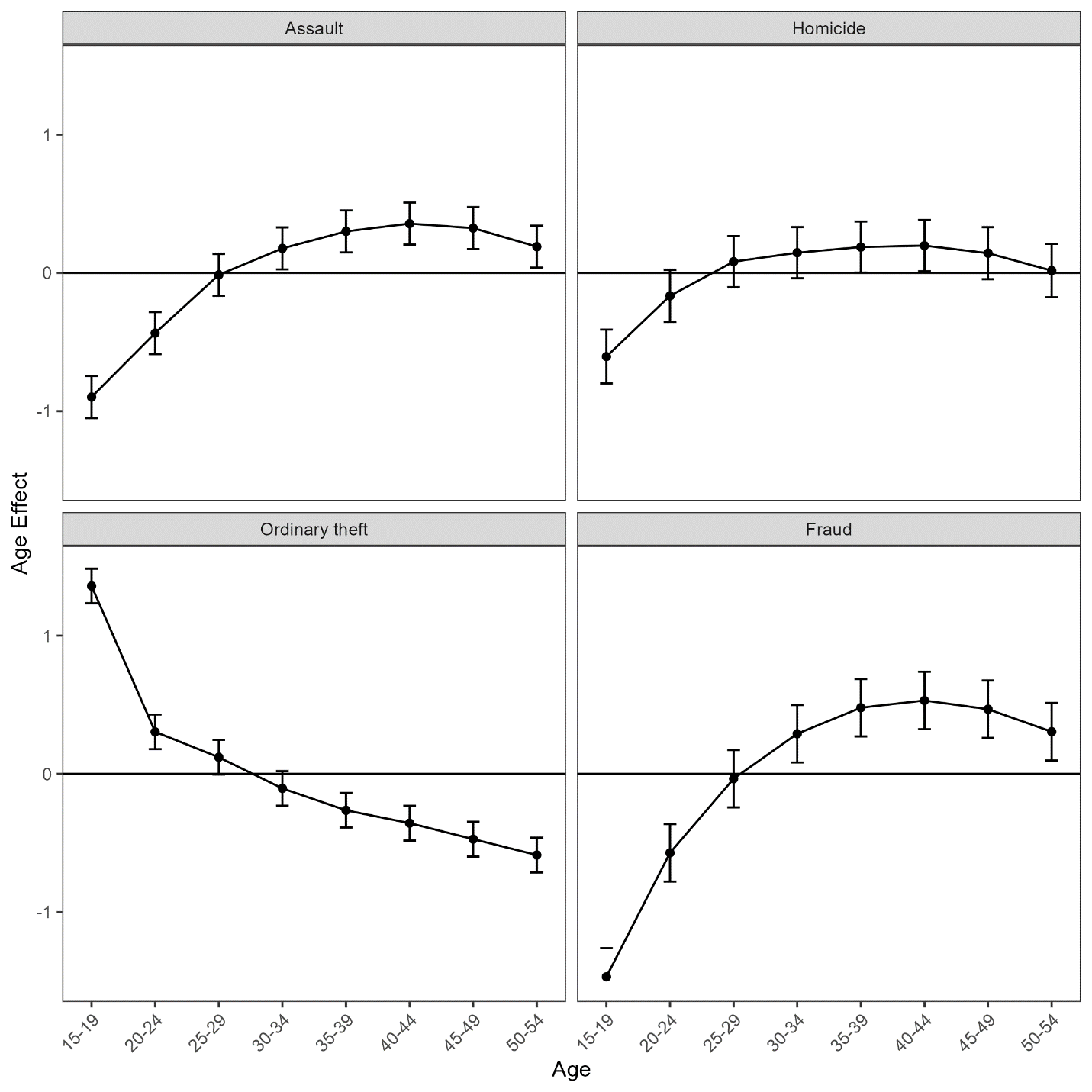


# C. Detailed model statistics and data visualization of the APC-I analysis.

## S1 Table 1. Estimated age and period main effects on arrest rates of different offenses in South Korea.

|  | Assault | | | Homicide | | | Ordinary Theft | | | Fraud | | |
| --- | --- | --- | --- | --- | --- | --- | --- | --- | --- | --- | --- | --- |
|  | Coef. | SE | Sig | Coef. | SE | Sig | Coef. | SE | Sig | Coef. | SE | Sig |
| Intercept | -6.039 | 0.002 | *** | -10.620 | 0.015 | *** | -6.368 | 0.002 | *** | -5.393 | 0.001 | *** |
| Age Main Effects |  |  |  |  |  |  |  |  |  |  |  |  |
| 15-19 | -0.949 | 0.007 | *** | -0.663 | 0.052 | *** | 1.352 | 0.003 | *** | -1.649 | 0.006 | *** |
| 20-24 | -0.443 | 0.005 | *** | -0.161 | 0.039 | *** | 0.298 | 0.004 | *** | -0.576 | 0.004 | *** |
| 25-29 | 0.001 | 0.004 |  | 0.113 | 0.035 | ** | 0.124 | 0.004 | *** | 0.011 | 0.003 | *** |
| 30-34 | 0.188 | 0.004 | *** | 0.193 | 0.034 | *** | -0.093 | 0.005 | *** | 0.314 | 0.002 | *** |
| 35-39 | 0.305 | 0.004 | *** | 0.232 | 0.034 | *** | -0.247 | 0.005 | *** | 0.486 | 0.002 | *** |
| 40-44 | 0.367 | 0.004 | *** | 0.218 | 0.036 | *** | -0.341 | 0.006 | *** | 0.554 | 0.002 | *** |
| 45-49 | 0.332 | 0.004 | *** | 0.113 | 0.042 | ** | -0.473 | 0.007 | *** | 0.509 | 0.003 | *** |
| 50-54 | 0.198 | 0.005 | *** | -0.045 | 0.049 |  | -0.620 | 0.008 | *** | 0.351 | 0.003 | *** |
|  |  |  |  |  |  |  |  |  |  |  |  |  |
| Period Main Effects |  |  |  |  |  |  |  |  |  |  |  |  |
| 1980-84 | -0.558 | 0.006 | *** | 0.030 | 0.045 |  | 0.163 | 0.006 | *** | -0.512 | 0.005 | *** |
| 1985-89 | -0.718 | 0.006 | *** | -0.005 | 0.044 |  | -0.052 | 0.006 | *** | -0.594 | 0.004 | *** |
| 1990-94 | -0.778 | 0.006 | *** | 0.078 | 0.039 | * | -0.420 | 0.007 | *** | -0.313 | 0.004 | *** |
| 1995-99 | -0.581 | 0.005 | *** | 0.103 | 0.037 | ** | -0.369 | 0.006 | *** | 0.324 | 0.003 | *** |
| 2000-04 | -0.382 | 0.005 | *** | 0.075 | 0.037 | * | -0.172 | 0.005 | *** | 0.326 | 0.003 | *** |
| 2005-09 | 0.730 | 0.003 | *** | -0.111 | 0.042 | ** | 0.100 | 0.004 | *** | 0.233 | 0.003 | *** |
| 2010-14 | 1.027 | 0.003 | *** | -0.001 | 0.038 |  | 0.396 | 0.004 | *** | 0.274 | 0.003 | *** |
| 2015-19 | 1.260 | 0.002 | *** | -0.169 | 0.041 | *** | 0.354 | 0.004 | *** | 0.261 | 0.003 | *** |
|  |  |  |  |  |  |  |  |  |  |  |  |  |
| Cohort | (See S3 Table 2) | | | | | | | | | | | |

Notes: All APC-I models are estimated with the sum-to-zero coding in R (rather than coding with a reference category), under which the main effect is interpreted as the deviation from the grand mean and the interaction term as the deviation from the main effects. Different from the conventional modeling approach that uses an age or period category as the reference group, the coefficients estimated using the sum-to-zero coding represent the deviation from the grand mean of all the observations. This approach also makes the interpretation of interaction terms easier as each coefficient of the interaction term represents the deviation from the expected values based on the main effects. Moreover, this approach also allows us to adjust for the arrest level differences across offenses—that is, we can compare the estimated coefficients across offense types as the mean differences across offense types are held constant in the model.

* p<.05; ** p<.01; *** p<.001. Because we use population data and we do not intend to make statistical inferences outside of the range of the study period of our data, statistical significance is included only as a reference. We focus on the effect sizes as they can be interpreted as population parameters rather than sample statistics

## S1 Table 2. Estimated inter-cohort deviations of different offenses in South Korea

|  | Assault | | | Homicide | | | Ordinary Theft | | | Fraud | | |
| --- | --- | --- | --- | --- | --- | --- | --- | --- | --- | --- | --- | --- |
| Cohort | Coef. | SE | Sig. | Coef. | SE | Sig. | Coef. | SE | Sig. | Coef. | SE | Sig. |
| 1930 | -0.286 | 0.020 | *** | -0.371 | 0.173 | * | -0.278 | 0.025 | *** | 0.170 | 0.011 | *** |
| 1935 | -0.221 | 0.013 | *** | -0.539 | 0.117 | *** | -0.290 | 0.016 | *** | 0.106 | 0.007 | *** |
| 1940 | -0.106 | 0.009 | *** | -0.472 | 0.084 | *** | -0.245 | 0.013 | *** | 0.076 | 0.005 | *** |
| 1945 | 0.079 | 0.007 | *** | -0.201 | 0.063 | ** | -0.093 | 0.011 | *** | 0.151 | 0.004 | *** |
| 1950 | 0.187 | 0.006 | *** | 0.011 | 0.048 |  | 0.039 | 0.008 | *** | 0.138 | 0.003 | *** |
| 1955 | 0.253 | 0.005 | *** | 0.219 | 0.038 | *** | 0.175 | 0.006 | *** | 0.142 | 0.003 | *** |
| 1960 | 0.123 | 0.004 | *** | 0.258 | 0.032 | *** | 0.137 | 0.005 | *** | -0.053 | 0.003 | *** |
| 1965 | -0.002 | 0.004 |  | 0.178 | 0.030 | *** | -0.013 | 0.004 | ** | -0.162 | 0.004 | *** |
| 1970 | -0.131 | 0.005 | *** | 0.243 | 0.033 | *** | 0.065 | 0.004 | *** | -0.117 | 0.004 | *** |
| 1975 | -0.296 | 0.006 | *** | 0.005 | 0.039 |  | -0.051 | 0.005 | *** | -0.286 | 0.005 | *** |
| 1980 | -0.160 | 0.006 | *** | -0.141 | 0.049 | ** | 0.011 | 0.005 | * | -0.221 | 0.004 | *** |
| 1985 | -0.166 | 0.006 | *** | -0.309 | 0.063 | *** | -0.161 | 0.005 | *** | -0.042 | 0.004 | *** |
| 1990 | 0.034 | 0.005 | *** | -0.317 | 0.084 | *** | -0.033 | 0.005 | *** | 0.113 | 0.006 | *** |
| 1995 | 0.395 | 0.006 | *** | -0.252 | 0.098 | ** | -0.118 | 0.006 | *** | 0.650 | 0.006 | *** |
| 2000 | 0.857 | 0.008 | *** | -0.242 | 0.166 |  | -0.353 | 0.007 | *** | 1.168 | 0.010 | *** |

Notes: * p<.05; ** p<.01; *** p<.001. Inter-cohort deviation represents a cohort’s average deviation from the predicted rate determined by age and period main effects. A positive inter-cohort deviation represents higher-than-expected arrest risks, whereas a negative inter-cohort deviation represents the opposite. A small inter-cohort deviation not significantly different from zero suggests no cohort effect.

## S1 Fig 3. Estimated period main effects and inter-cohort deviations of different offenses in South Korea.

1. Period Plots^1^


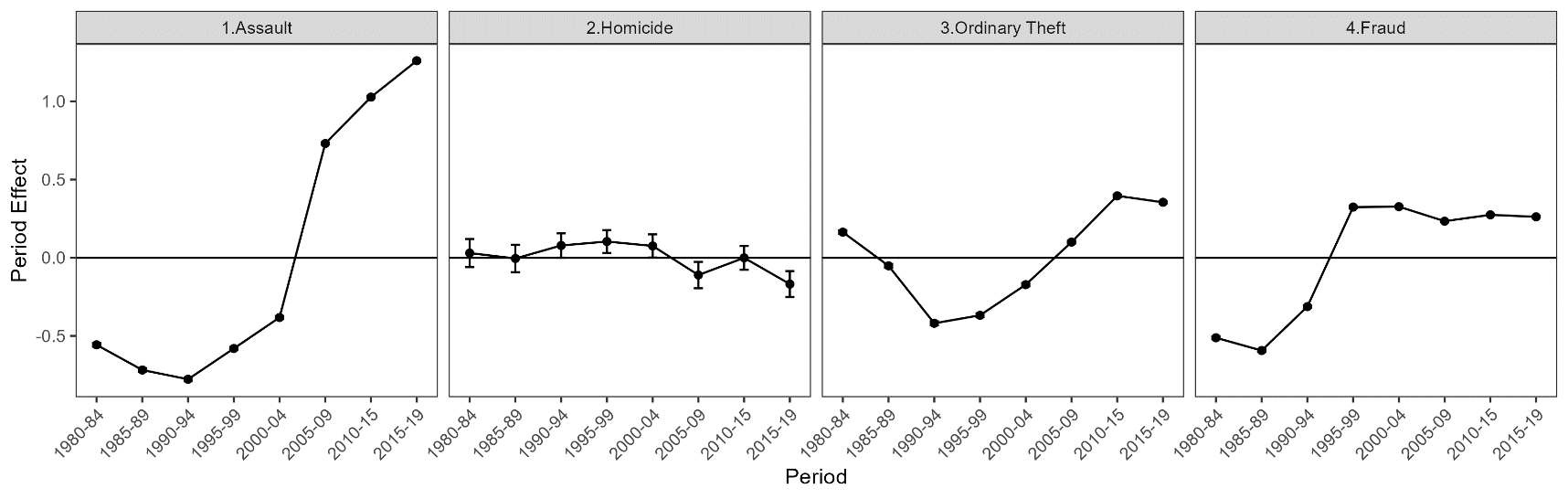


1. Cohort Plots^2^

**
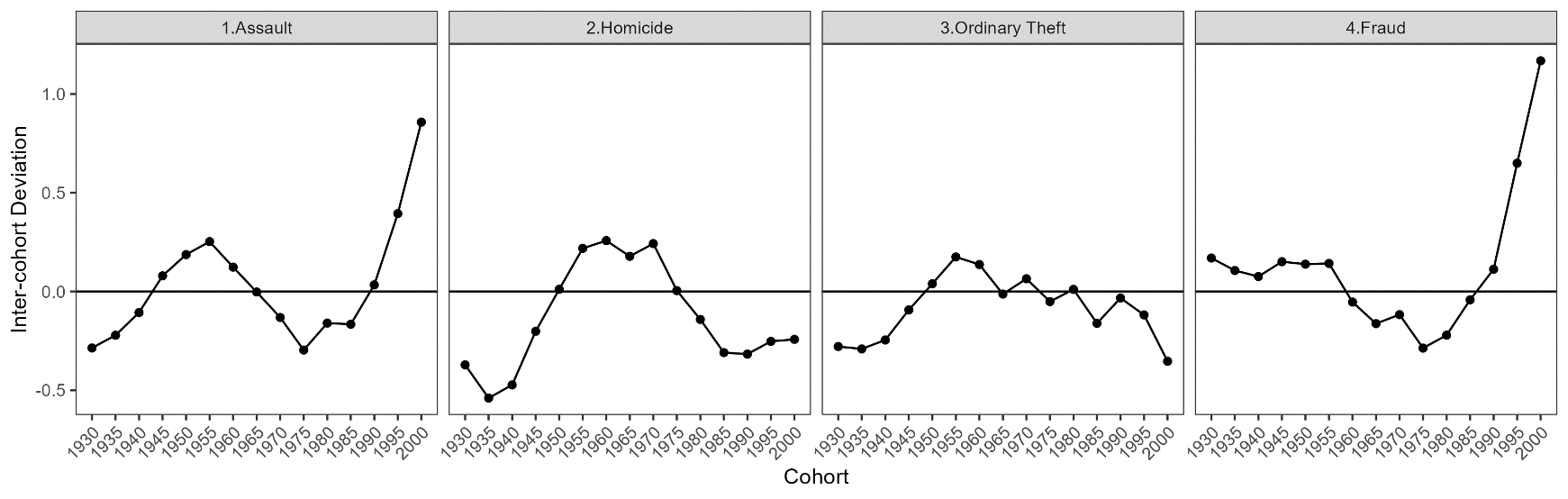
**

Notes: 1. For the period plots, the horizontal solid line represents zero deviation from the global mean (intercept of the APC-I model). Points above the zero line represent positive deviations from the global mean, suggesting higher-than-expected risks of arrest; points below the zero line represent negative deviations from the global mean, suggesting lower-than-expected risks of arrest. 2. For the cohort plots, the horizontal solid line in each plot represents no deviation from the predicted arrest rate determined by age and period main effects. Points that fall on the zero line represent no cohort effects (i.e., age and period main effects are sufficient to determine the arrest rate). Points above the line indicate cohorts with higher arrest rates than the predicted values determined by age and period main effects. Points below the zero line indicate cohorts with lower arrest rates than the predicted values determined by age and period main effects.

# D. Supplemental analysis with post-adolescence data.

## S1 Fig 4. Estimated age main effects on arrest rates of different offense types with *post-adolescence data (age 20+)*, controlling for period and cohort main effects.


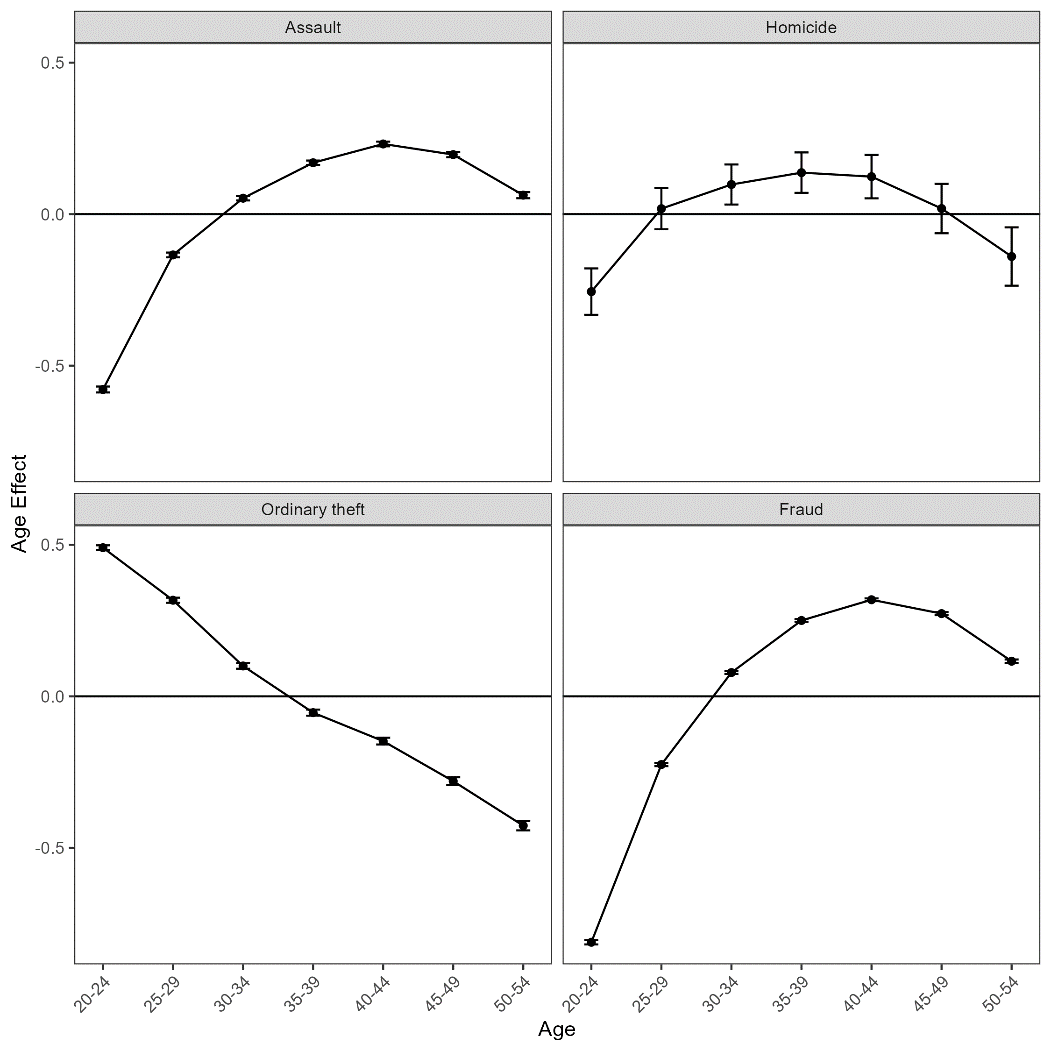


## S1 Fig 5. Predicted age-crime distributions by offense types and models across historical periods with *post-adolescence data* (age 20+).


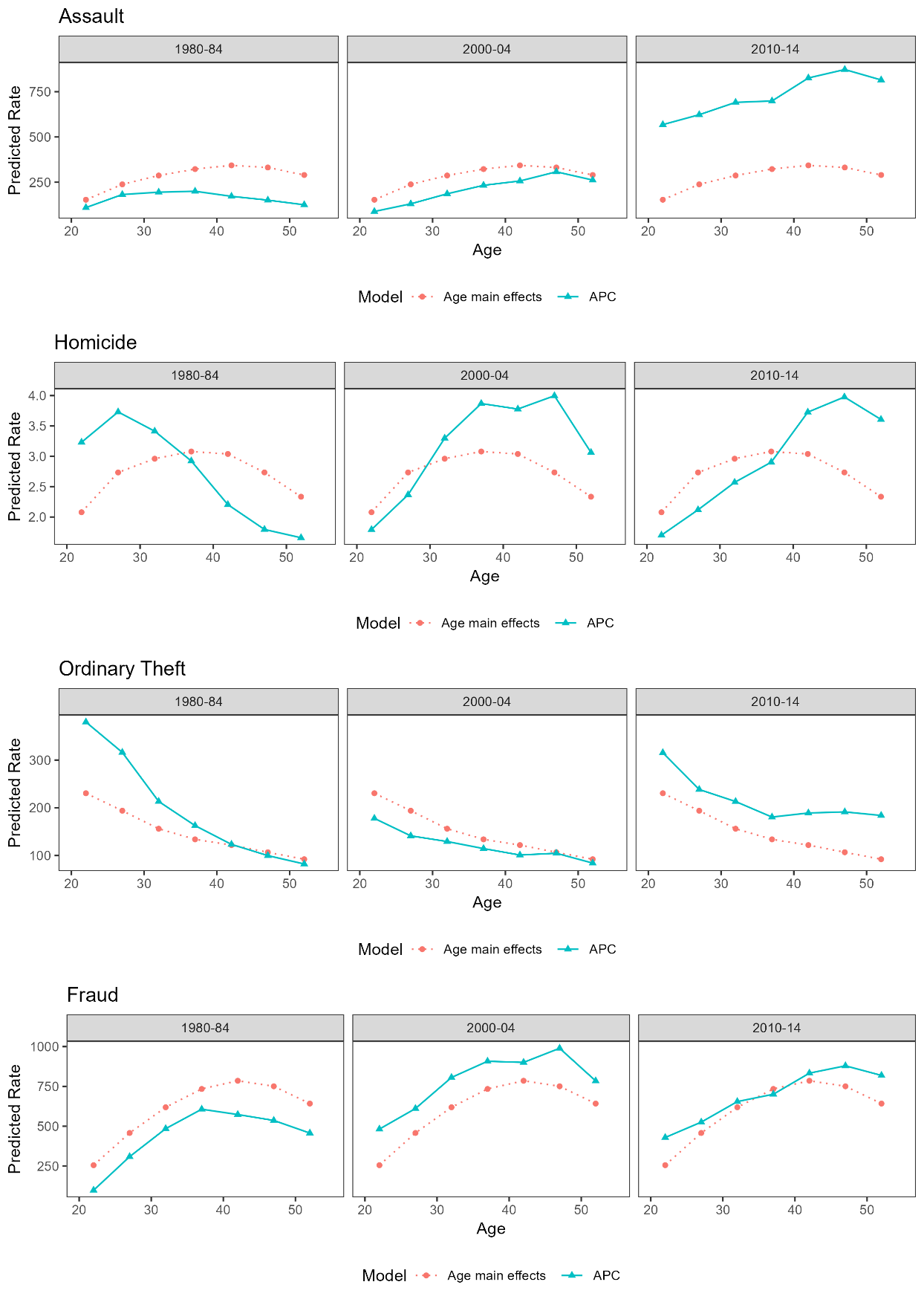


Notes: The red dotted curve in each plot represents the predicted age curve with age main effects only, controlling for period and cohort effects, which we also define as the average time-invariant curve. The blue solid curve represents the predicted age curve of the APC model (i.e., with age, period, and cohort effects). The deviation of the blue solid curve from the red dotted curve indicates how period and cohort effects modify the average age-arrest curve across different periods.

# E. References of the technical appendix

1. Thornberry T, Krohn M. Comparison of self-report and official data for measuring crime. Washington, DC: National Academies Press; 2003 pp. 43–94. doi:10.17226/10581

2. Department of Justice. The Nation’s two crime measures. Washington DC: Department of Justice; 2004.

3. Lu Y, Luo L. Cohort Variation in U.S. Violent Crime Patterns from 1960 to 2014: An Age–Period–Cohort-Interaction Approach. J Quant Criminol. 2021;37: 1047–1081. doi:10.1007/s10940-020-09477-3

4. Lu Y, Steffensmeier D. Stability or Change in Age-Crime Relation in Taiwan, 1980–2019: Age-Period-Cohort Assessment. Asian J Criminol. 2023;18: 433–458. doi:10.1007/s11417-023-09412-y

5. O’Brien RM. Relative cohort size and age-specific crime rates: An age-period-relative-cohort-size model. Criminology. 1989;27: 57–78. doi:10.1111/j.1745-9125.1989.tb00863.x

6. Rennó Santos M, Lu Y, Fairchild RE. Age, Period and Cohort Differences Between the Homicide Trends of Canada and the United States. Br J Criminol. 2021;61: 389–413. doi:10.1093/BJC/AZAA080

7. Steffensmeier D, Streifel C, Shihadeh ES. Cohort Size and Arrest Rates Over the Life Course: The Easterlin Hypothesis Reconsidered. Am Sociol Rev. 1992;57: 306–314. doi:10.2307/2096237

8. Vogel M, Thompson KJ, Messner SF. The Enduring Influence of Cohort Characteristics on Race-Specific Homicide Rates. Soc Forces. 2019;99: 1–30. doi:10.1093/sf/soz127

9. You M. Social Change, Cohort Effects, and Dynamics of the Age–Crime Relationship: Age and Crime in South Korea from 1967 to 2011. J Quant Criminol. 2023 [cited 16 Oct 2023]. doi:10.1007/s10940-023-09579-8

10. Holly S, Jones N. House prices since the 1940s: Cointegration, demography and asymmetries. Econ Model. 1997;14: 549–565. doi:10.1016/S0264-9993(97)00009-6

11. Lidwall U, Marklund S. Trends in long-term sickness absence in Sweden 1992–2008: the role of economic conditions, legislation, demography, work environment and alcohol consumption. Int J Soc Welf. 2011;20: 167–179. doi:10.1111/j.1468-2397.2010.00744.x

12. Rudnytskyi O, Levchuk N, Wolowyna O, Shevchuk P, Kovbasiuk (Savchuk) A. Demography of a man-made human catastrophe: The case of massive famine in Ukraine 1932-1933. Can Stud Popul Arch. 2015;42: 53–80. doi:10.25336/P6FC7G

13. Weden MM, Peterson CE, Miles JN, Shih RA. Evaluating Linearly Interpolated Intercensal Estimates of Demographic and Socioeconomic Characteristics of U.S. Counties and Census Tracts 2001–2009. Popul Res Policy Rev. 2015;34: 541–559. doi:10.1007/s11113-015-9359-8

14. McNeil DR, Trussell TJ, Turner JC. Spline interpolation of demographic data. Demography. 1977;14: 245–252. doi:10.2307/2060581

15. Bergstrom T, Lam D. Recovering event histories by cubic spline interpolation. Math Popul Stud. 1989;1: 327–355. doi:10.1080/08898488909525283

16. Fritsch FN, Carlson RE. Monotone Piecewise Cubic Interpolation. SIAM J Numer Anal. 1980;17: 238–246. doi:10.1137/0717021

17. Kostaki A, Panousis V. Expanding an abridged life table. Demogr Res. 2001;5: 1–22.

18. Luo L, Hodges JS. The Age-Period-Cohort-Interaction Model for Describing and Investigating Inter-cohort Deviations and Intra-cohort Life-course Dynamics. Sociol Methods Res. 2022;51: 1164–1210.

19. Hobcraft J, Menken J, Preston S. Age, Period, and Cohort Effects in Demography: A Review. Popul Index. 1982;48: 4–43. doi:10.2307/2736356

20. Ryder NB. The cohort as a concept in the study of social change. Am Sociol Rev. 1965;30: 843–861. doi:Doi 10.2307/2090964

21. Rydberg J, Carkin DM. Utilizing Alternate Models for Analyzing Count Outcomes. Crime Delinquency. 2017;63: 61–76. doi:10.1177/0011128716678848

22. Wasserstein RL, Lazar NA. The ASA statement on p-values: Context, process, and purpose. Am Stat. 2016;70: 129–133. doi:10.1080/00031305.2016.1154108

23. Alexander N. What’s more general than a whole population? Emerg Themes Epidemiol. 2015;12: 11. doi:10.1186/s12982-015-0029-4

24. Berk RA, Western B, Weiss RE. Statistical Inference for Apparent Populations. Sociol Methodol. 1995;25: 421–458. doi:10.2307/271073

25. Lu Y, Luo L, Santos MR. Social Change and Race-Specific Homicide Trajectories: An Age-Period-Cohort Analysis. J Res Crime Delinquency. 2022; 00224278221129886. doi:10.1177/00224278221129886
